# Comparison of admission glycemic variability and glycosylated hemoglobin in predicting major adverse cardiac events among type 2 diabetes patients with heart failure following acute ST-segment elevation myocardial infarction

**DOI:** 10.2478/jtim-2024-0006

**Published:** 2024-05-21

**Authors:** Xiuxiu Yang, Gong Su, Tao Zhang, Hongxia Yang, Hong Tao, Xin Du, Jianzeng Dong

**Affiliations:** Department of Cardiology, Beijing Anzhen Hospital, Capital Medical University, Beijing 100029, China; Department of Cardiology, Aerospace Center Hospital, Peking University Aerospace School of Clinical Medicine, Beijing 100049, China; Department of Endocrinology, Beijing Anzhen Hospital, Capital Medical University, Beijing 100029, China

**Keywords:** glycemic variability, glycosylated hemoglobin, acute ST-segment elevation myocardial infarction, major adverse cardiac events

## Abstract

**Background and Objectives:**

Hyperglycemia is associated with adverse outcomes in patients with acute myocardial infarction (AMI) as well as in patients with heart failure. However, the significance of admission glycemic variability (GV) in predicting outcomes among diabetes patients with heart failure (HF) following acute ST-segment elevation myocardial infarction (ASTEMI) remains unclear. This study aims to explore the prognostic value of admission GV and admission glycosylated hemoglobin (HbA1c) levels in individuals diagnosed with type 2 diabetes and HF following ASTEMI.

**Methods:**

We measured GV and HbA1c upon admission in 484 consecutive patients diagnosed with type 2 diabetes and HF following ASTEMI. GV, indicated as the mean amplitude of glycemic excursions (MAGE), was assessed utilizing a continuous glucose monitoring system (CGMS). admission MAGE values were categorized as < 3.9 or ≥ 3.9 mmol/L, while HbA1c levels were classified as < 6.5 or ≥ 6.5%. Participants were followed up prospectively for 12 months. The relationship of admission MAGE and HbA1c to the major adverse cardiac event (MACE) of patients with type 2 diabetes and HF following ASTEMI was analyzed.

**Results:**

Among the 484 enrolled patients, the occurrence of MACE differed significantly based on MAGE categories (< 3.9 *vs*. ≥ 3.9 mmol/L), with rates of 13.6% and 25.3%, respectively (*P* = 0.001). While MACE rates varied by HbA1c categories (< 6.5 *vs*. ≥ 6.5%) at 15.7% and 21.8%, respectively (*P* = 0.086). Patients with higher MAGE levels exhibited a notably elevated risk of cardiac mortality and an increased incidence of HF rehospitalization. The Kaplan-Meier curves analysis demonstrated a significantly lower event-free survival rate in the high MAGE level group compared to the low MAGE level group (log-rank test, *P* < 0.001), while HbA1c did not exhibit a similar distinction. In multivariate analysis, high MAGE level was significantly associated with incidence of MACE (hazard ratio 3.645, 95% CI 1.287–10.325, *P* = 0.015), whereas HbA1c did not demonstrate a comparable association (hazard ratio 1.075, 95% CI 0.907-1.274, *P* = 0.403).

**Conclusions:**

Elevated admission GV emerges as a more significant predictor of 1-year MACE in patients with type 2 diabetes and HF following ASTEMI, surpassing the predictive value of HbA1c.

## Introduction

Hyperglycemia is associated with adverse outcomes in patients with acute myocardial infarction (AMI) as well as those with heart failure (HF).^[1,2]^ Evidence suggests that abnormal blood glucose, evaluated through glycosylated hemoglobin (HbA1c) levels, may serve as a prognostic factor not only in patients with AMI but also in those with HF.^[3,4]^ However, more acute disturbances in glucose metabolism may also exert a negative impact on patient outcomes. Glycemic variability (GV), also known as blood glucose fluctuation or glycemic excursions, is a parameter that reflects changes in blood glucose levels independently of FPG, postprandial blood glucose, and HbA1c. It represents another crucial indicator of glycemic control. Studies have demonstrated that HbA1c cannot directly reflect GV. The same HbA1c level may exhibit diverse daily glucose profiles. Patients with identical HbA1c levels may exhibit different GV, and there is no significant correlation between the two.^[5,6]^ Historically, HbA1c has been served as the primary indicator for assessing glycemic control and utilized in primary and secondary prevention of coronary heart disease. However, recent research indicates that, in comparison to HbA1c, GV may exhibit a more closely correlation with the severity of both microvascular and macrovascular atherosclerosis. GV appears to potential function as an independent risk factor for cardiovascular complications in individuals with diabetes.^[7,8,9,10,11^] Nevertheless, whether admission GV and admission HbA1c levels hold prognostic significance for patients with type 2 diabetes and HF following acute ST-segment elevation myocardial infarction (ASTEMI) remains unclear. Therefore, the aim of this study is to investigate the independent prognostic value of GV assessed by a continuous glucose monitoring system (CGMS) upon admission and admission HbA1c levels in type 2 diabetes patients with HF following ASTEMI.

## Methods

### Study population

This is a single-center prospective follow-up study. Consecutive type 2 diabetic patients with HF following ASTEMI admitted to the cardiology department of Beijing Anzhen Hospital of Capital Medical University between February 2020 and January 2023 were selected. The inclusion criteria were: (1) confirmed admission diagnosis of ASTEMI, HF and Type 2 diabetes mellitus (T2DM), (2) admission glucose level < 16.7 mmol/L, and (3) without diabetic ketosis or nonketotic hyperosmolar coma. ST-segment elevation myocardial infarction was defined as complaints of chest pain with electrocardiogram (ECG) signs compatible with AMI (ST-segment elevation > 2 mm in precordial leads and > 1 mm in limb leads).

Myocardial infarction was defined as acute if the time elapsed between the first symptom and admission was 72 h or less. T2DM was diagnosed according to the American Diabetes Association criteria or the use of insulin or glucose-lowering medication. HF includes both heart failure with reduced ejection fraction (HFrEF) and heart failure with mildly reduced ejection fraction (HFmrEF). HF was diagnosed according to 2021 ESC Guidelines for the Diagnosis and Treatment of Acute and Chronic Heart Failure. The exclusion criteria were: (1) severe noncardiac disease with expected survival was less than 1 year and unwillingness to participate, (2) with Cushing’s syndrome, hyperthyroidism and other diseases affecting glucose metabolism, (3) with other diseases that need to use glucocorticoid or other medications affecting glucose metabolism, (4) with chronic heart failure, chronic obstructive pulmonary disease or cardiomyopathy, (5) with infections, severe liver and kidney diseases, tumors or autoimmune diseases. A patient could only be included once. Thus, 484 patients with complete data were included in the final analysis. Patients were grouped based on mean amplitude of glycemic excursions (MAGE) levels into < 3.9 mmol/L and ≥ 3.9 mmol/L categories, following the criteria outlined in the Chinese Guidelines for Clinical Application of Blood Glucose Monitoring (2021 Edition) and the Chinese Guidelines for Clinical Application of Continuous Glucose Monitoring (2017 Edition). The study protocol was approved beforehand by the Medical Ethics Committee of Beijing Anzhen Hospital of Capital Medical University and the procedures followed were in accordance with the institutional guidelines. The study complied with the declaration of Helsinki and informed consent was obtained from all patients.

### Continuous glucose monitoring system

All patients were equipped with CGMS (Medtronic MiniMed, USA), and were monitored for 72 consecutive hours after admission. A CGMS sensor was inserted into the subcutaneous abdominal fat tissue, calibrated according to the standard Medtronic MiniMed operating guidelines. During CGMS monitoring, patients checked their blood glucose level with a self-monitoring of blood glucose (SMBG) device (Medisafe Mini, Terumo, Japan) at least 4 times per day. Then, the SMBG data and time of each meal were entered into the CGMS. After monitoring for 72 hours, the recorded data were downloaded into a personal computer for analysis of the glucose profile and glucose excursion parameters with MiniMed Solutions software. Analysis was limited to the data obtained from the intermediate 48 hours of recording to avoid bias due to insertion and removal of the CGMS or insufficient stability of the monitoring system. Since measurable range of glucose by CGMS was mechanically limited from 2.2 to 22.2 mmol/L, the case showing the data out of this range was excluded from the study.^[12]^

### Transthoracic echocardiography

All patients underwent echocardiography within the first 24 hours of admission. The assessments of transthoracic echocardiography (GE Vivid 7 cardiovascular ultrasound Doppler system, USA) were conducted by the designated experienced sonographer for all patients. The measurements included the left ventricular ejection fraction (LVEF) and left ventricular end-diastolic diameter (LVEDD) using the biplane Simpson’s method. Each measurement was averaged over three cardiac cycles for accurate representation.

### Biochemical investigations

Blood samples were collected after overnight fasting and stored at-70°C prior to analysis. FPG, serum creatinine, total cholesterol (TC), low-density lipoprotein cholesterol (LDL-C), high-density lipoprotein-cholesterol (HDL-C), triglyceride (TG), creatinine levels and high-sensitive C-reactive protein (hs-CRP) were measured by automatic biochemical analyzer (Hitachi 747, Tokyo, Japan). Serum concentration of HbA1c was determined by high-performance liquid chromate graphic method using an automatic HbA1c analyzer (Tosoh HLC-723G7; Tosoh Corporation, Tokyo, Japan).

### Follow-up

Patients were followed up prospectively for about 12 months. During the follow-up period, incidences of major adverse cardiac event (MACE) were registered, including new-onset myocardial infarction, Re-hospitalization for acute heart failure, and cardiac death. All MACE data were adjudicated by an experienced cardiovascular physician blinded to clinical details and outcomes.

### Statistical analysis

All statistical analyses were performed by using SPSS for Windows 27.0 (SPSS Inc, Chicago, IL, USA). Data are presented as frequencies and percentages for categorical variables and mean ± SD for continuous variables, unless otherwise indicated. Differences between two groups were assessed by using the Chi-square and unpaired t-tests. Correlation between continuous variables was determined by Spearman correlation coefficients. Admission MAGE was included as a continuous and as a categorized (< 3.9 or ≥ 3.9 mmol/L) variable. Admission HbA1c levels were also included as continuous and categorized (< 6.5 and ≥6.5%) variables. Kaplan-Meier survival curve analysis was employed to delineate the proportional risk of MACE based on admission MAGE and HbA1c values. The log-rank test was subsequently conducted to evaluate disparities between high and low MAGE levels, as well as high and low HbA1c levels. To ascertain the independent contribution to MACE, multivariate regression analysis was conducted. A value of *P* < 0.05 was considered statistically significant.

## Results

### Baseline characteristics

During the study period, 484 patients with complete data were enrolled (6 patients were removed from study for severe dysglycemia during CGMS monitoring period; 8 patients were excluded from study for incomplete follow-up data). Mean age was 63.6 ± 9.3 years, 62.6% were male. Baseline characteristics of patient groups based on MAGE and HbA1c level are shown in Table 1 and 2, respectively. Participants were treated conservatively (15.6%), with PCI (76.6%) or with CABG (7.8%). The GRACE risk score ranged from 78 to 234 with a mean of 149 ± 38. The correlation of GRACE score with MAGE or HbA1c was significant (Spearman *r* = 0.348, *P* < 0.001; *r* = 0.163, *P* = 0.019).

**Table 1:** Baseline characteristics in patients according to MAGE level

Characteristics	MAGE (mmol/L)		*P*

	< 3.9	≥ 3.9	
*n*	286	198	
Age (years)	62.56 ± 8.91	65.04 ± 9.67	0.089
Males	177 (61.9)	126 (63.6)	0.696
Risk factors			
Smoking	117 (40.9)	90 (45.5)	0.320
BMI (kg/m^2^)	26.19 ± 2.12	26.68 ± 2.35	0.232
Hypertension	175 (61.2)	129 (65.2)	0.375
LVEF (%)	45.06 ± 10.01	40.76 ± 10.93	0.011
eGFR (ml/min/1.73 m^2^)	74.67 ± 26.14	66.34 ± 18.97	0.041
TC (mmol/L)	4.71 ± 1.23	4.82 ± 1.42	0.297
TG (mmol/L)	2.08 ± 1.08	2.31 ± 1.16	0.064
FBG (mmol/L)	7.94 ± 2.68	9.03 ± 2.77	< 0.001
HbA1c (%)	6.67 ± 1.45	7.42 ± 1.49	< 0.001
Duration of DM (months)	30.48 ± 40.32	48.21 ± 50.76	< 0.001
Previous CAD	70 (24.5)	75 (37.9)	0.002
Medications			
Aspirin	186 (65.0)	134 (67.7)	0.546
Beta-blocker	123 (43.0)	93 (47.0)	0.389
Oral anti-hyperglycemic	247 (86.4)	182 (91.9)	0.058
Insulin	85 (29.7)	78 (39.4)	0.027
ACEI or ARB	124 (43.4)	90 (45.5)	0.648
ARNI	26 (9.1)	20 (10.1)	0.709
Statin	232 (81.1)	165 (83.3)	0.533
Diuretic	41 (14.3)	32 (16.2)	0.581
GRACE score	138 ± 36	154 ± 39	< 0.001

MAGE: the mean amplitude of glycemic excursions; BMI: body mass index; LVEF: left ventricular ejection fraction; eGFR: estimated glomerular filtration rate; TC: total cholesterol; TG: triglyceride; FBG: fasting blood glucose; HbA1c: hemoglobin A1c; DM: diabetes mellitus; CAD: coronary artery disease; ACEI: angiotensin converting enzyme inhibitor; ARB: Angiotensin Receptor Blocker; ARNI: Angiotensin Receptor-Neprilysin Inhibitor; GRACE: the global registry of acute coronary events. Data are mean ± SD or number (%).

**Table 2:** Baseline characteristics in patients according to HbA1c level

Characteristics	HbA1c (%)		*P*

	< 6.5	≥ 6.5	
*n*	268	216	
Age (years)	62.27 ± 8.98	65.19 ± 9.48	0.041
Males	161 (60.1)	142 (65.7)	0.200
Risk factors			
Smoking	112 (41.8)	95 (44.0)	0.628
BMI (kg/m^2^)	26.24 ± 2.15	26.57 ± 2.31	0.635
Hypertension	167 (62.3)	137 (63.4)	0.801
LVEF (%)	44.01 ± 10.38	42.42 ± 10.42	0.059
eGFR (ml/min/1.73 m^2^)	75.12 ± 24.32	66.48 ± 22.17	0.033
TC (mmol/L)	4.67 ± 1.24	4.86 ± 1.39	0.102
TG (mmol/L)	1.99 ± 1.06	2.40 ± 1.18	0.038
FBG (mmol/L)	7.63 ± 2.30	9.32 ± 2.89	< 0.001
MAGE (mmol/L)	2.67 ± 1.21	4.06 ± 1.33	< 0.001
Duration of DM (months)	27.03 ± 37.01	51.01 ± 53.25	< 0.001
Previous CAD	71 (26.5)	74 (34.3)	0.064
Medications			
Aspirin	169 (63.1)	151 (69.9)	0.114
Beta-blocker	124 (46.3)	92 (42.6)	0.419
Oral anti-hyperglycemic	230 (85.8)	199 (92.1)	0.030
Insulin	69 (25.7)	94 (43.5)	< 0.001
ACEI or ARB	112 (41.8)	102 (47.2)	0.232
ARNI	23 (8.6)	23 (10.6)	0.441
Statin	213 (79.55)	184 (85.2)	0.104
Diuretic	37 (13.8)	36 (16.7)	0.382
GRACE score	140 ± 34	150 ± 37	0.022

HbA1c: hemoglobin A1c; BMI: body mass index; LVEF: left ventricular ejection fraction; eGFR: estimated glomerular filtration rate; TC: total cholesterol; TG: triglyceride; FBG: fasting blood glucose; MAGE: the mean amplitude of glycemic excursions; DM: diabetes mellitus; CAD: coronary artery disease; ACEI: angiotensin converting enzyme inhibitor; ARB: Angiotensin Receptor Blocker; ARNI: Angiotensin Receptor-Neprilysin Inhibitor; GRACE: the global registry of acute coronary events. Data are mean ± SD or number (%).

### Incidences of MACE

**Figure 1:**
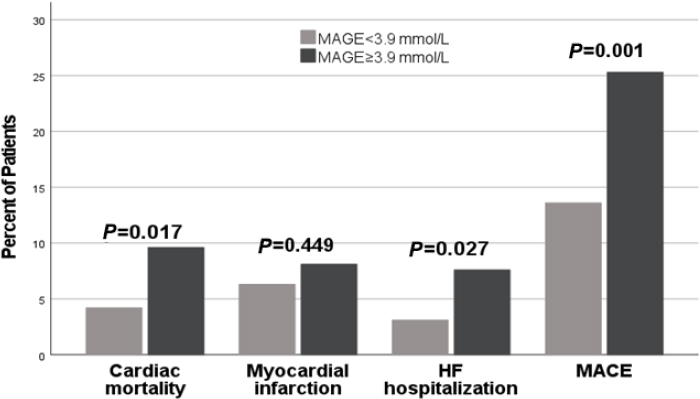
Incidence of major adverse cardiac event (MACE) after 1-year follow-up in relation to MAGE levels. Patients with a higher MAGE level had significantly higher cardiac mortality, the rate of HF rehospitalization and incidence of all MACE.

**Figure 2:**
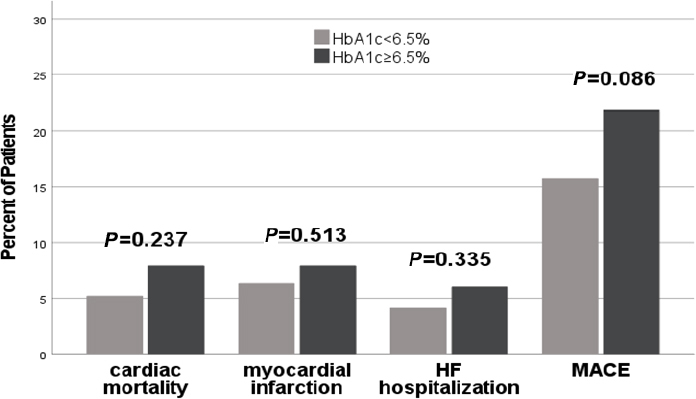
Incidence of major adverse cardiac event (MACE) after 1-year follow-up in relation to HbA1c levels. There are no significant differences of adverse cardiovascular events rates between different HbA1c level groups (all *P* > 0.05).

**Figure 3:**
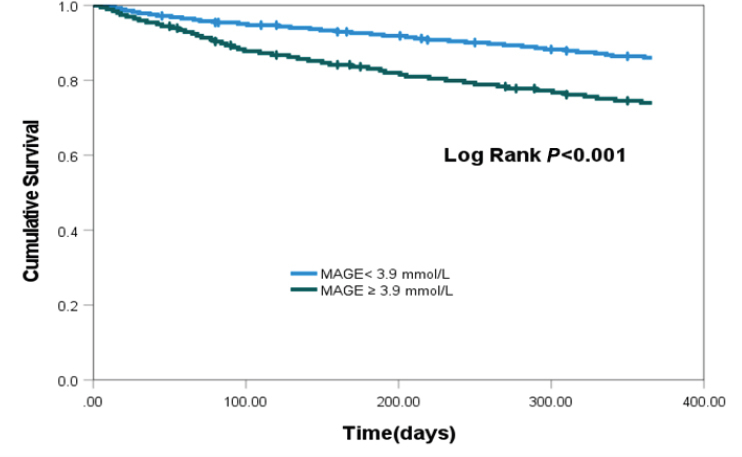
Kaplan-Meier event-free survival curves for freedom from major adverse cardiac event (MACE) in two patient groups by MAGE levels. The event-free survival rate was significantly lower in the high MAGE level group (log-rank test, *P* < 0.001).

**Figure 4:**
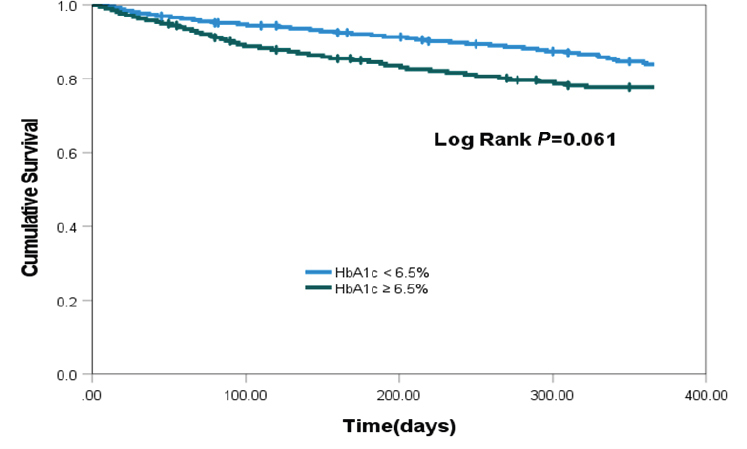
Kaplan-Meier event-free survival curves for freedom from major adverse cardiac event (MACE) in two patient groups by HbA1c levels. There is not significant lower event-free survival rate in high HbA1c level patients (log-rank test, *P* = 0.061).

At the end of 1-year follow-up, 31 patients had died (6.4%) for cardiac causes, 34 patients had new-onset myocardial infarction (7.0%), and 24 patients were re-hospitalized for acute heart failure (5.0%). Among patients diagnosed with type 2 diabetes and HF following ASTEMI, a notably elevated incidence of MACE was observed in those presenting with MAGE level ≥ 3.9 mmol/L compared to their counterparts with MAGE levels < 3.9 mmol/L (25.3 *vs*. 13.6%; *P* = 0.001). There were no statistically significant differences in the rates of adverse cardiovascular events observed between patients with Hemoglobin A1c (HbA1c) levels ≥ 6.5% and those with HbA1c levels < 6.5% (21.8% *vs*. 15.7%, *P* = 0.086). Patients with higher MAGE levels demonstrated a significantly increased incidence of cardiac mortality in comparison to those with lower MAGE levels (9.6% *vs*. 4.2%, *P* = 0.017). Patients with heightened MAGE levels exhibited a significantly increased incidence of HF rehospitalization when contrasted with individuals characterized by lower MAGE levels (7.6% *vs*. 3.1%, *P* = 0.027) (Figure 1). No statistically significant differences were observed in the rates of cardiac mortality, new-onset myocardial infarction, and HF rehospitalization among patients with varying levels of admission HbA1c (Figure 2). Kaplan-Meier survival curves for patient groups by MAGE are shown in Figure 3; those for HbA1c are in Figure 4.

### Multivariate analysis

To investigate the associations between MAGE, HbA1c level and incidences of MACE with respect to baseline characteristics, multivariable analysis was employed. The included variables encompassed age, gender, and all variables that exhibited statistically significant differences across MAGE or HbA1c categories. These variables comprised LVEF, estimated Glomerular Filtration Rate (eGFR), fasting blood glucose levels, duration of diabetes mellitus (DM), history of previous coronary artery disease (CAD), TG levels, usage of oral anti-hyperglycemic agents, and insulin therapy. The independent predictors of MACE were age, LVEF, previous CAD, and MAGE (Table 3). Admission HbA1c level was not significantly associated with MACE (HR 1.075, 95% CI: 0.907–1.274, *P* = 0.403).

**Table 3:** Multivariate analysis of determinants of MACE

Independent variables	B	S. E.	Wald	*P*	Exp (B)	95% CI for Exp (B)
Constant	-6.291	1.339	22.061	< 0.001	0.002	
Age (years)	1.673	0.749	4.987	0.026	1.317	(1.227, 1.428)
LVEF	-0.519	0.248	4.396	0.036	0.595	(0.366, 0.967)
Previous CAD	0.217	0.095	5.216	0.022	1.243	(1.031, 1.497)
MAGE	1.293	0.531	5.928	0.015	3.645	(1.287, 10.325)

LVEF: left ventricular ejection fraction; CAD: coronary artery disease.

## Discussion

Elevated blood glucose levels upon admission are frequently observed in patients presenting with AMI, serving as a robust predictor of survival and an elevated risk of MACE in individuals, irrespective of their T2DM status.^[13,14]^ Additionally, hyperglycemia is significantly associated with adverse outcomes in patients diagnosed with heart failure. There is very limited research on the impact of dysglycaemia at admission on the prognosis of heart failure patients. Furthermore, there is a notable absence of studies specifically addressing the prognostic implications of admission dysglycaemia in diabetic patients developing heart failure after acute myocardial infarction. HbA1c serves as a convenient marker reflecting the longterm glycometabolic status. Elevated levels of HbA1c are associated with an augmented cardiovascular risk in patients. There are two types of disorders in glucose metabolism: sustained chronic hyperglycemia and glucose fluctuation. It has been observed that patients with similar average glucose or HbA1c levels can have very different glucose fluctuations. Acute fluctuations in glucose levels appear to exert more deleterious effects than sustained hyperglycemia in the progression of cardiovascular complications, as both upward and downward changes in glucose activate oxidative stress.^[15,16,17]^ We explored the relationship between GV, HbA1c and one-year MACE in individuals with type 2 diabetes and HF following ASTEMI. Our investigation revealed that elevated MAGE emerged as a robust and independent predictor of an increased risk of MACE in type 2 diabetes patients with HF following ASTEMI, whereas HbA1c did not exhibit a similar predictive capacity.

Significant differences in baseline characteristics were observed based on levels of MAGE or HbA1c. Patients with elevated MAGE or HbA1c levels exhibited a higher prevalence of cardiovascular risk factors, including an extended duration of DM, impaired left ventricular function, kidney dysfunction, hyperlipemia, and a history of previous CAD. A discernible correlation was observed between GRACE risk scores and both MAGE and HbA1c. These findings suggest that individuals with diabetes and HF following ASTEMI characterized by more severe glycometabolic disorders may be associated with poorer outcomes.

An increasing body of evidence suggests that, despite similar HbA1c levels, there may still be variations in the occurrence and progression of diabetic complications. In recent years, both basic and clinical studies have shown that the occurrence and development of diabetic complications are not only related to the mean blood glucose level (persistence), but also closely related to the fluctuation of blood glucose (stability). GV may serve as an important parameter for addressing potential clinical problems in diabetes. To explore the damage caused by GV to target organs, numerous basic and clinical research studies have been conducted. Temelkova-Kurktschiev *et al*. identified that postchallenge glucose excursions are more strongly associated with carotid intima-media thickness than FPG and HbA1c levels and substantially modify the risk for atherosclerosis estimated by HbA1c alone, in a cohort at risk for diabetes and in the early diabetes stage.^[18]^ In our prior investigation, we identified GV as a significant contributing factor to the severity of coronary artery disease, independently of the average blood glucose level.^[19]^ The Verona Diabetes Study reported that fasting GV independently predicts mortality in patients with T2DM.^[20]^ Several studies have concluded that GV serves as a substantial predictor of mortality in critically ill patients, independent of mean glucose levels and the severity of illness.^[21,22]^ In this study, a one-year follow-up revealed a significantly increased incidence of MACE, cardiac mortality, and re-hospitalization for HF among patients with higher MAGE levels. These findings suggest a potential association between elevated glucose fluctuations and the heightened risk of future adverse cardiovascular events in diabetes patients with HF following ASTEMI. Multivariable analysis further demonstrated that, in the diabetic population with HF following ASTEMI, MAGE emerged as an independent predictor of MACE. This association remained significant even after adjusting for all variables that exhibited significant differences between MAGE or HbA1c categories, whereas HbA1c did not demonstrate similar independent predictive capability. Acute hyperglycemia serves as a prevalent acute adrenergic manifestation in response to stress and is observed in myocardial infarction. Elevated catecholamine levels precipitate diminished insulin secretion and heightened insulin resistance during such instances.^[23]^ While stress-induced hyperglycemia provides partial elucidation for the correlation between admission GV and outcomes, it is crucial to recognize that glycemic excursion itself can independently contribute to deleterious effects. Although stress-induced hyperglycemia provides partial elucidation for the correlation between admission GV and outcomes, glycemic excursion itself can also be harmful. Quagliaro *et al*. compared the damage to endothelial cells under two abnormal blood glucose conditions: sustained hyperglycemia and glucose fluctuation. Human umbilical vein endothelial cells (HUVECs) were incubated for 14 days in media containing different glucose concentrations: 5 mmol/L, 20 mmol/L, or a daily alternating 5 or 20 mmol/L glucose. The studies performed show that both intermittent and high glucose concentrations stimulate apoptosis, but intermittent glucose concentrations appear to worsen the proapoptotic effects of high glucose on HUVECs.^[24,25]^ Some studies suggest that sustained hyperglycemia increases the levels of nitrotyrosine, 8-hydroxydeoxyguanosine (8-OHdG), and apoptosis. However, these effects are more pronounced in the context of glucose fluctuation. Protein kinase C (PKC) levels are elevated in both conditions, but particularly in glucose fluctuation. Inhibitors of PKC, such as bisindolylmaleimide-I and LY379196, have been shown to normalize nitrotyrosine levels, reduce 8-OHdG concentration, and decrease cell apoptosis. Glucose fluctuation also stimulates the overproduction of reactive oxygen species (ROS) through PKC-dependent activation of NAD (P) H oxidase, leading to increased cellular apoptosis at the mitochondrial transport chain level.^[24,25]^ Glycemic excursion may also be an important mediator in inflammatory responses. Studies indicate that glucose fluctuations can activate nuclear factor-ϰB and PKC pathway, leading to a greater expression of the adhesion molecules (ICAM-1, VCAM-1, and E-selectin), and promoting interleukin-6 (IL-6) production, and excess formation of advanced glycation endproducts than stable high glucose.^[26]^ Furthermore, profound glycemic disorders may exert an unfavorable impact on sympathetic dysfunction, a factor intricately linked with the mortality and morbidity of cardiovascular diseases.^[27]^

While both HbA1c and GV may exhibit associations with an adverse prognosis, our study reveals a greater significance attributed to increased MAGE. In our analysis, the relatively unclear association between HbA1c and MACE may be attributed to a restricted number of patients and a relatively short follow-up duration in present study. Elevated HbA1c levels signify prolonged glucose dysregulation, reflecting long-term glycemic control. In contrast, heightened glucose fluctuation not only serves as an indicator of glucose dysregulation but also serves as a manifestation of stress and overall compromised health. Carmen Wong found that both the level of hyperglycaemia and cortisol levels on admission are predictive for the subsequent abnormal glucose tolerance development in hyperglycaemic AMI patients. However, hyperglycaemia in patients who are more unwell (i. e. higher cortisol) reflects the stressed state rather than underlying glucose intolerance. Conversely, if the patient is less sick (i. e. lower cortisol), hyperglycaemia is more likely to reflect underlying glucose intolerance.^[28]^ A discernible association between HbA1c levels and long-term outcomes in AMI patients is evident following a 3.3-year follow-up period.^[29]^ Discrepancies observed in predictive power may be attributed to the duration of follow-up. While HbA1c levels may demonstrate limited predictive capacity for short-term prognosis in diabetic patients with HF following ASTEMI, its association with long-term prognosis appears to be more robust.

Ongoing extensive debate persists regarding the status of glycemic excursion as an independent risk factor for cardiovascular complications, distinct from HbA1c.^[30,31]^ Siegelaar *et al*. conducted a reanalysis of data from the HEART2D study, revealing that targeting post-prandial glucose to reduce intraday glycemic excursion may not confer benefits in mitigating adverse cardiovascular events in AMI patients.^[32]^ Nevertheless, it is crucial to note that the HEART2D study was not specifically designed to assess the impact of glycemic excursion on the risk of MACE, and the MAGE levels were not significantly different between contrasting groups in the study. Additionally, the method employed to calculate GV from self-measured blood glucose profiles may lack precision. Overall, more rigorously designed studies are warranted to elucidate whether glycemic excursion plays a significant role in the prognostication of AMI.

## Study limitations

The relatively small sample size may limit the power of subgroup comparisons to detect significant differences for selected variables. Due to the absence of data on microvascular complications, these risk factors were not incorporated into the analysis. Despite maintaining patients’ antihyperglycemic therapy and avoiding glucose infusion during CGMS observation, factors such as variations in diet, physical activity, and emotional stress, which can influence glucose fluctuations, could not be entirely mitigated. Furthermore, routine tests for diabetes detection were not conducted, potentially resulting in overlooked cases of diabetes. However, if the observed association between glycemic excursion and MACE were attributed to undiagnosed diabetes, a more pronounced correlation with HbA1c and outcomes would have been anticipated.

## Conclusions

In patients with type 2 diabetes and HF following ASTEMI, admission MAGE emerges as a potentially crucial predictor of MACE, whereas HbA1c did not exhibit similar predictive significance. In the glycemic metabolism of patients with type 2 diabetes and HF following ASTEMI, it appears that acute glucose excursion holds greater predictive significance for 1-year outcomes compared to long-term derangements in glucose metabolism. The findings of this study provide support for the perspective that glycemic excursion should be considered as a focal point for the treatment of glycemic disorders observed in patients with type 2 diabetes and HF following ASTEMI. Additional research is warranted to ascertain the benefits of pharmacologic therapy targeting glycemic excursion in patients with type 2 diabetes and HF following ASTEMI regarding the prognosis of this high-risk patient population.

## References

[1] Schmitt VH, Hobohm L, Münzel T, Wenzel P, Gori T, Keller K (2021). Impact of diabetes mellitus on mortality rates and outcomes in myocardial infarction. Diabetes Metab.

[2] Baldia PH, Marx N, Schütt KA. (2020). Diabetes and Heart failure. Dtsch Med Wochenschr.

[3] Gustafsson I, Kistorp CN, James MK, Faber JO, Dickstein K, Hildebrandt PR, OPTIMAAL Study Group (2007). Unrecognized glycometabolic disturbance as measured by hemoglobin A1c is associated with a poor outcome after acute myocardial infarction. Am Heart J.

[4] He J, Xi Y, Lam H, Du K, Chen D, Dong Z (2023). Effect of Intensive Glycemic Control on Myocardial Infarction Outcome in Patients with Type 2 Diabetes Mellitus: A Systematic Review and Meta-Analysis. J Diabetes Res.

[5] Huang L, Pan Y, Zhou K, Liu H, Zhong S (2023). Correlation Between Glycemic Variability and Diabetic Complications: A Narrative Review. Int J Gen Med.

[6] Kusunoki Y, Konishi K, Tsunoda T, Koyama H (2022). Significance of Glycemic Variability in Diabetes Mellitus. Intern Med.

[7] Yapanis M, James S, Craig ME, O’Neal D, Ekinci EI (2022). Complications of Diabetes and Metrics of Glycemic Management Derived From Continuous Glucose Monitoring. J Clin Endocrinol Metab.

[8] Wang T, Zhang X, Liu J (2022). Long-Term Glycemic Variability and Risk of Cardiovascular Events in Type 2 Diabetes: A Meta-Analysis. Horm Metab Res.

[9] Alfieri V, Myasoedova VA, Vinci MC, Rondinelli M, Songia P, Massaiu I (2021). The Role of Glycemic Variability in Cardiovascular Disorders. Int J Mol Sci.

[10] Zhang L, Li F, Liu HH, Zhang ZY, Yang F, Qian LL (2022). Glycaemic variability and risk of adverse cardiovascular events in acute coronary syndrome. Diab Vasc Dis Res.

[11] Martinez M, Santamarina J, Pavesi A, Musso C, Umpierrez GE (2021). Glycemic variability and cardiovascular disease in patients with type 2 diabetes. BMJ Open Diabetes Res Care.

[12] Lazar S, Ionita I, Reurean-Pintilei D, Timar B (2023). How to Measure Glycemic Variability? A Literature Review. Medicina (Kaunas).

[13] Thoegersen M, Josiassen J, Helgestad OK, Berg Ravn H, Schmidt H, Holmvang L (2020). The association of diabetes and admission blood glucose with 30-day mortality in patients with acute myocardial infarction complicated by cardiogenic shock. Eur Heart J Acute Cardiovasc Care.

[14] Djupsjö C, Kuhl J, Andersson T, Lundbäck M, Holzmann MJ, Nyström T. (2022). Admission glucose as a prognostic marker for all-cause mortality and cardiovascular disease. Cardiovasc Diabetol.

[15] Monnier L, Mas E, Ginet C, Michel F, Villon L, Cristol JP (2006). Activation of oxidative stress by acute glucose fluctuations compared with sustained chronic hyperglycemia in patients with type 2 diabetes. JAMA.

[16] Chang Chih-Min, Hsieh Ching-Jung, Huang Ju-Chun, Huang I-Chin (2012). Acute and chronic fluctuations in blood glucose levels can increase oxidative stress in type 2 diabetes mellitus. Acta Diabetol.

[17] Valente T, Arbex AK (2021). Glycemic variability, oxidative stress, and impact on complications related to type 2 diabetes mellitus. Curr Diabetes Rev..

[18] Temelkova-Kurktschiev TS, Koehler C, Henkel E, Leonhardt W, Fuecker K, Hanefeld M (2000). Postchallenge plasma glucose and glycemic spikes are more strongly associated with atherosclerosis than fasting glucose or HbA1c level. Diabetes Care.

[19] Su G, Mi S, Tao H, Li Z, Yang H, Zheng H (2011). Association of glycemic variability and the presence and severity of coronary artery disease in patients with type 2 diabetes. Cardiovasc Diabetol.

[20] Muggeo M, Zoppini G, Bonora E, Brun E, Bonadonna RC, Moghetti P (2000). Fasting plasma glucose variability predicts 10-year survival of type 2 diabetic patients: the Verona Diabetes Study. Diabetes Care.

[21] Dossett LA, Cao H, Mowery NT, Dortch MJ, Morris JM Jr, May AK (2008). Blood glucose variability is associated with mortality in the surgical intensive care unit. Am Surg.

[22] Krinsley JS (2008). Glycemic variability: a strong independent predictor of mortality in critically ill patients. Crit Care Med.

[23] Takada JY, Ramos RB, Roza LC, Avakian SD, Ramires JA, Mansur Ade P. (2012). In-hospital death in acute coronary syndrome was related to admission glucose in men but not in women. Cardiovasc Diabetol.

[24] Quagliaro L, Piconi L, Assaloni R, Martinelli L, Motz E, Ceriello A (2003). Intermittent high glucose enhances apoptosis related to oxidative stress in human umbilical vein endothelial cells: the role of protein kinase C and NAD(P)H-oxidase activation. Diabetes.

[25] Piconi L, Quagliaro L, Assaloni R, Da Ros R, Maier A, Zuodar G (2006). Constant and intermittent high glucose enhances endothelial cell apoptosis through mitochondrial superoxide overproduction. Diabetes Metab Res Rev.

[26] Quagliaro L, Piconi L, Assaloni R, Da Ros R, Maier A, Zuodar G (2005). Intermittent high glucose enhances ICAM-1, VCAM-1 and E-selectin expression in human umbilical vein endothelial cells in culture: the distinct role of protein kinase C and mitochondrial superoxide production. Atherosclerosis.

[27] Takei Y, Tomiyama H, Tanaka N, Yamashina A (2007). Close relationship between sympathetic activation and coronary microvascular dysfunction during acute hyperglycemia in subjects with atherosclerotic risk factors. Circ J.

[28] Carmen Wong KY, Wong V, Ho JT, Torpy DJ, McLean M, Cheung NW (2010). High cortisol levels in hyperglycaemic myocardial infarct patients signify stress hyperglycaemia and predict subsequent normalization of glucose tolerance. Clin Endocrinol (Oxf).

[29] Timmer JR, Hoekstra M, Nijsten MW, van der Horst IC, Ottervanger JP, Slingerland RJ (2011). Prognostic value of admission glycosylated hemoglobin and glucose in nondiabetic patients with ST-segment-elevation myocardial infarction treated with percutaneous coronary intervention. Circulation.

[30] Kilpatrick ES, Rigby AS, Atkin SL (2010). For debate. Glucose variability and diabetes complication risk: we need to know the answer. Diabet Med.

[31] Lipska KJ, Venkitachalam L, Gosch K, Kovatchev B, Van den Berghe G, Meyfroidt G (2012). Glucose variability and mortality in patients hospitalized with acute myocardial infarction. Circ Cardiovasc Qual Outcomes.

[32] Siegelaar SE, Kerr L, Jacober SJ, Devries JH (2011). A decrease in glucose variability does not reduce cardiovascular event rates in type 2 diabetic patients after acute myocardial infarction: a reanalysis of the HEART2D study. Diabetes Care.

